# Free-Running Cardiac and Respiratory Motion-Resolved Imaging: A Paradigm Shift for Managing Motion in Cardiac MRI?

**DOI:** 10.3390/diagnostics14171946

**Published:** 2024-09-03

**Authors:** Robert J. Holtackers, Matthias Stuber

**Affiliations:** 1 Department of Diagnostic and Interventional Radiology, Lausanne University Hospital (CHUV) and University of Lausanne (UNIL), Rue de Bugnon 46, 1011 Lausanne, Switzerland; 2 Department of Radiology & Nuclear Medicine, Maastricht University Medical Center, P. Debyelaan 25, 6229 HX Maastricht, The Netherlands; 3 Cardiovascular Research Institute Maastricht (CARIM), Maastricht University, Universiteitssingel 50, 6200 MD Maastricht, The Netherlands; 4 Center for Biomedical Imaging (CIBM), EPFL AVP CP CIBM Station 6, 1015 Lausanne, Switzerland

**Keywords:** free-running, cardiac MRI, CMR, 5D, motion-resolved, self-gating, free-breathing

## Abstract

Cardiac magnetic resonance imaging (MRI) is widely used for non-invasive assessment of cardiac morphology, function, and tissue characteristics due to its exquisite soft-tissue contrast. However, it remains time-consuming and requires proficiency, making it costly and limiting its widespread use. Traditional cardiac MRI is inefficient as signal acquisition is often limited to specific cardiac phases and requires complex view planning, parameter adjustments, and management of both respiratory and cardiac motion. Recent efforts have aimed to make cardiac MRI more efficient and accessible. Among these innovations, the free-running framework enables 5D whole-heart imaging without the need for an electrocardiogram signal, respiratory breath-holding, or complex planning. It uses a fully self-gated approach to extract cardiac and respiratory signals directly from the acquired image data, allowing for more efficient coverage in time and space without the need for electrocardiogram gating, triggering, navigators, or breath-holds. This review provides a comprehensive overview of the free-running framework, detailing its history, concepts, recent improvements, and clinical applications.

## 1. Introduction

Cardiac magnetic resonance imaging (MRI) has been widely adopted for the non-invasive assessment of cardiac morphology, function, and tissue characteristics because of its superior soft-tissue contrast compared to other imaging modalities. Although continued technical advancements and developments have pushed the envelope for expanding clinical applications, cardiac MRI remains time-consuming and requires proficient operators, contributing to its overall costs and limiting its more widespread global use.

Cardiac MRI is time-consuming because of the inherently low signals that are sampled in MRI on the one hand and due to the relatively complex patient setup and cardiac view planning procedures on the other hand. Inefficiency is further exacerbated if signal acquisition is limited to selected time points within the cardiac and/or respiratory cycle. The relatively complex preparations and cardiac view planning, combined with the need to manage both respiratory and cardiac motion, and therefore define additional parameters, make cardiac MRI not only time-consuming but also proficiency-demanding.

An increasing effort is being undertaken to make cardiac MRI more efficient, less user-dependent, and faster—also in the interest of disseminating cardiac MRI beyond large urban academic centres. Among these efforts is the fully automated free-running framework (FRF), enabling highly efficient cardiac and respiratory motion-resolved whole-heart imaging without the need for electrocardiogram (ECG) electrode positioning, respiratory breath-holding, navigators, and complex cardiac view planning. This review aims to provide a comprehensive overview of the possibilities of the FRF, including its history and background, basic concepts and mechanisms, recent developments and improvements, various (clinical) applications, and general recommendations. Finally, conclusions on the current evidence and limitations are drawn, and new avenues for future research are discussed.

## 2. History and Rationale of Self-Gated Cardiac MRI

**Figure 1:**
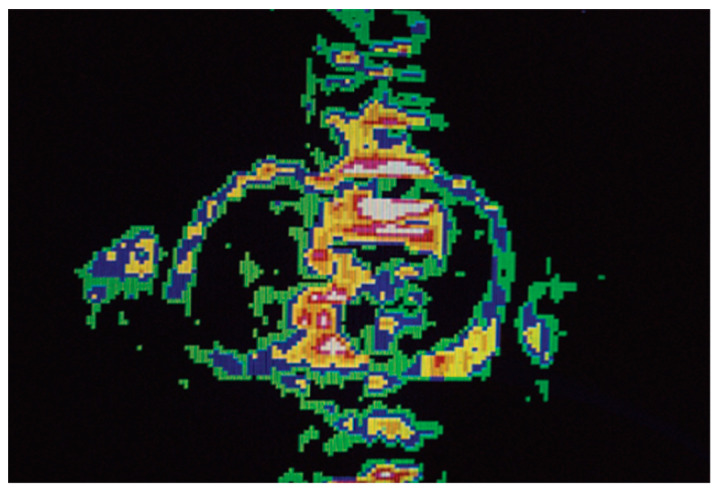
One of the first MRI images of the heart was acquired in a volunteer using the Aberdeen NMR imager in 1979, before the spin-warp breakthrough. Although the contours of the body and some internal structures can be recognized, severe artefacts caused by cardiac motion were present preventing its clinical use. Image courtesy of Bill Edelstein.

Cardiac motion significantly complicates magnetic resonance imaging of the heart as it adversely affects image quality if not adequately accounted for. If motion occurs, inconsistencies among the acquired profiles in k-space and/or unwanted signal phase accrual during signal readout are among the sources of artefacts. Therefore, the suppression of motion artefacts in cardiac MRI is the most challenging, interesting, and rewarding field of active engineering research. In fact, on one of the very first MR images of the heart that was acquired in Aberdeen by Bill Edelstein and his team in the late 1970s, heart motion was immediately recognized as one of the sources of artefacts (Figure 1) [1]. In the ensuing years, with the advent of gradient hardware that permitted shortened repetition times, ECG triggering, and k-space segmentation, breath-holding became the mainstay for motion artefact suppression in functional and anatomical cardiovascular MRI [2].

Although prospective ECG gating enables signal acquisition during a specific cardiac phase to ‘freeze’ cardiac motion, and respiratory breath-hold commands can be provided to do the same for respiratory motion, these mechanisms are inherently inefficient and therefore time-consuming. In addition, temporal resolution and spatial image resolution were governed by a patient-dependent duration and quality of the breath-hold. These shortcomings were rate-limiting for 3D imaging with large volumetric coverage, where image acquisition with high temporal and spatial resolution became impractical using breath-holding to suppress respiratory motion artefacts. During the past two decades, an abundance of work has focussed on addressing both the cardiac and respiratory motion problems for improved performance of cardiovascular MRI.

With the development of respiratory navigators by Ehman et al., this hurdle could be removed and free-breathing 3D imaging of the heart with larger volumetric coverage became available [3]. However, navigator localization and parameter settings remained operator-dependent, and individual respiratory patterns led to various degrees of navigator efficiency and ultimately unpredictable scan times, which complicated the integration of navigator-gated free-breathing 3D scans into routine clinical protocols.

Interestingly, the concept of cardiac self-gating (SG) emerged for 2D imaging around that time. In their seminal work, Larson et al. demonstrated that the cardiac signal can not only be extracted from continuously acquired k-space profiles but also be used to retrospectively bin data according to their position in the cardiac cycle without the need for an ECG [4]. The insights gained from this work stimulated Stehning et al. to exploit SG for the respiratory rather than for the cardiac signal to replace respiratory navigators with a respiratory SG concept [5]. Using 3D radial imaging during free-breathing and a repetitively acquired superior-inferior (SI) profile in k-space, respiratory SG was enabled. This helped minimize operator dependence, scan time was no longer affected by the patient’s ability to hold their breath or their respiratory pattern, and whole-heart 3D imaging during free breathing in just a few minutes of acquisition time became available. This concept was then refined by Piccini et al., who extended the ECG-triggered 3D radial readout with a phyllotaxis pattern and refined the respiratory signal extraction algorithm for improved image quality [6,7]. The well-defined and almost constant image acquisition duration of 9 min permitted an easy integration of the sequence into a clinical protocol, just during the wait time between perfusion imaging and late enhancement imaging [8].

Despite this progress, data were still acquired ECG-triggered and thus only during a short interval of quiescence typically in mid-diastole, which is not only highly inefficient but also involves time-consuming ECG lead placement and occasional repositioning of the electrodes in case any artefactual signal overlaid to the ECG is too strong. The first problem can be solved through a continuous, uninterrupted 3D radial golden-angle data collection where the ECG is not used for triggering—but still recorded for gating during reconstruction—as demonstrated by Coppo et al. [9]. When applying the above-mentioned respiratory motion correction algorithm that was developed for respiratory SG [5], 3D cine frames can be reconstructed, and the duty cycle improves 15-fold. Subsequently, and to further increase image quality, Feng et al. proposed the reconstruction of Coppo’s free-running data using their extra-dimensional golden-angle radial sparse parallel (XD-GRASP) algorithm, with which respiratory and cardiac resolved 3D data with excellent image quality could be reconstructed [10,11]. Considering that three spatial and two temporal dimensions are involved, the technique is often referred to as 5D. Nevertheless, this prompted interest in applying compressed sensing (CS) reconstruction algorithms to free-running 3D data. Coming back to the second shortcoming mentioned above and remembering the already cited work on cardiac SG by Larson et al. [4], it appears obvious that the SG signal extracted from the repetitively acquired SI profile should not only be exploited for respiratory but also cardiac motion. Considering that it is acquired approximately every 70 ms, mechanical contraction and relaxation of the heart can be resolved using this temporal resolution as no exact ECG pattern needs to be reconstructed from this signal. Spectral analysis and filtering of that signal permit the extraction of both respiratory and cardiac signature curves that, in turn, can be used to bin the acquired data according to their respiratory and cardiac position prior to a CS reconstruction.

This ‘total self-gating’ concept was first reported by Di Sopra et al. and formed the basis for complete free-running cardiac and respiratory motion-resolved 5D whole-heart MRI [12]. Please note, however, that free-running cardiac MRI can be performed using a variety of methods, including both non-Cartesian (spiral phyllotaxis [6,12], radial stack-of-stars [13,14,15], and 3D radial kooshball [10,16,17,18]) and Cartesian (MUSIC [19,20], centric reordering [21], and CASPR [22,23]) trajectories. In this review, we will focus on work that either originated from or expanded upon the original description of the FRF employing a 3D radial trajectory and total SG, as first reported by Di Sopra and colleagues.

## 3. The Fully Automated Cardiac Free-Running Framework

With the work of Di Sopra et al., the FRF was established as a push-button solution for 5D whole-heart MRI with a high isotropic spatial resolution (1.0–2.0 mm^3^) and high temporal resolution (15–30 phases per cardiac cycle). This FRF consists of a total SG approach extracting both cardiac and respiratory signals directly from the acquired image data, an automated reconstruction pipeline, and excellent MPR opportunities for the resulting dynamic 3D images, thereby offering significant advantages over conventional cardiac MRI. In the following sections, we will discuss the key parts of this FRF, including the acquisition, reconstruction, and advantages over conventional cardiac MRI.

### 3.1. Acquisition

**Figure 2:**
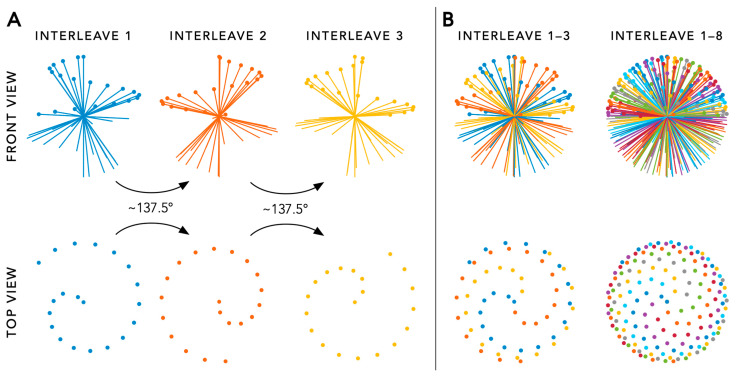
Panel (**A**) Schematic overview of 3D radial sampling using a spiral phyllotaxis readout pattern (also known as an interleave). When the pre-defined number of radial spokes of a single interleave has been acquired, the next interleave will start which is rotated about the golden angle (~137.5°) from the previous one. Panel (**B**) With an increasing number of interleaves, an increasingly dense 3D kooshball of data points is obtained. After a pre-defined number of interleaves, the free-running acquisition is finished.

The FRF utilizes a sequence which continuously acquires 3D radial k-space lines, also known as radial spokes, with each passing through the centre of k-space. All these radial spokes can be segmented into consecutive interleaves that each follow a phyllotaxis pattern and are rotated by the golden angle (~137.5°) from the previous one (Figure 2A) [6]. Two important parameters that define the phyllotaxis trajectory are the number of radial spokes acquired per interleave (also referred to as the number of segments), and the total number of interleaves needed to complete data collection. The multiplication of these two parameters results in the total number of radial spokes to be acquired in the sequence. With each radial spoke passing through the centre of k-space, a 3D kooshball is acquired and an image with an isotropic spatial resolution and isotropic field-of-view is typically generated (Figure 2B). The (isotropic) spatial resolution is usually set between 1.0 and 2.0 mm, where a higher resolution requires an increased number of interleaves to obtain sufficient image data for reconstruction. The readout of the radial spokes can be performed using either balanced steady-state free-precession (bSSFP) or regular gradient echo (GRE), of which the former is preferred at 1.5 T given its increased signal while the latter is the method of choice at 3 T to prevent banding artefacts.

**Figure 3:**
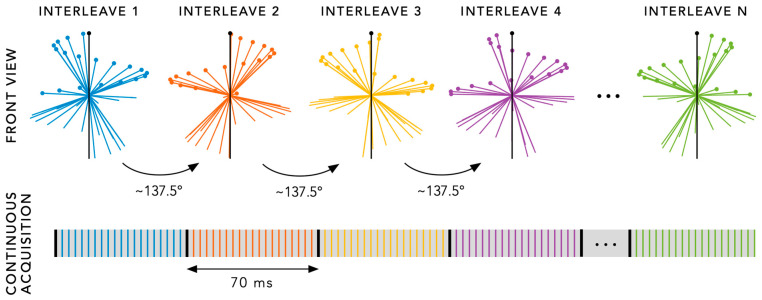
A schematic overview of enabling self-gating (SG) in 3D radial MRI by inserting a radial spoke in the superior-inferior direction at the start of each interleaf. Due to the continuous acquisition, an SG signal is obtained periodically as defined by the number of spokes per interleave (i.e., segments) and the repetition time (TR). With a TR of 3.5 ms and 20 segments per interleave (as shown), an SG signal is acquired every 70 ms and can be used to extract cardiac and respiratory motion information for total SG.

To enable total SG, an additional radial spoke in the SI direction (SI spoke) is periodically inserted at the beginning of each interleaf for subsequent extraction of the cardiac and respiratory SG signals (Figure 3). With typical sequence parameters, such as a repetition time of 3.5 ms and 20 segments (including the first SI spoke), an SG signal is acquired every 20 × 3.5 = ~70 ms.

### 3.2. Reconstruction

**Figure 4:**
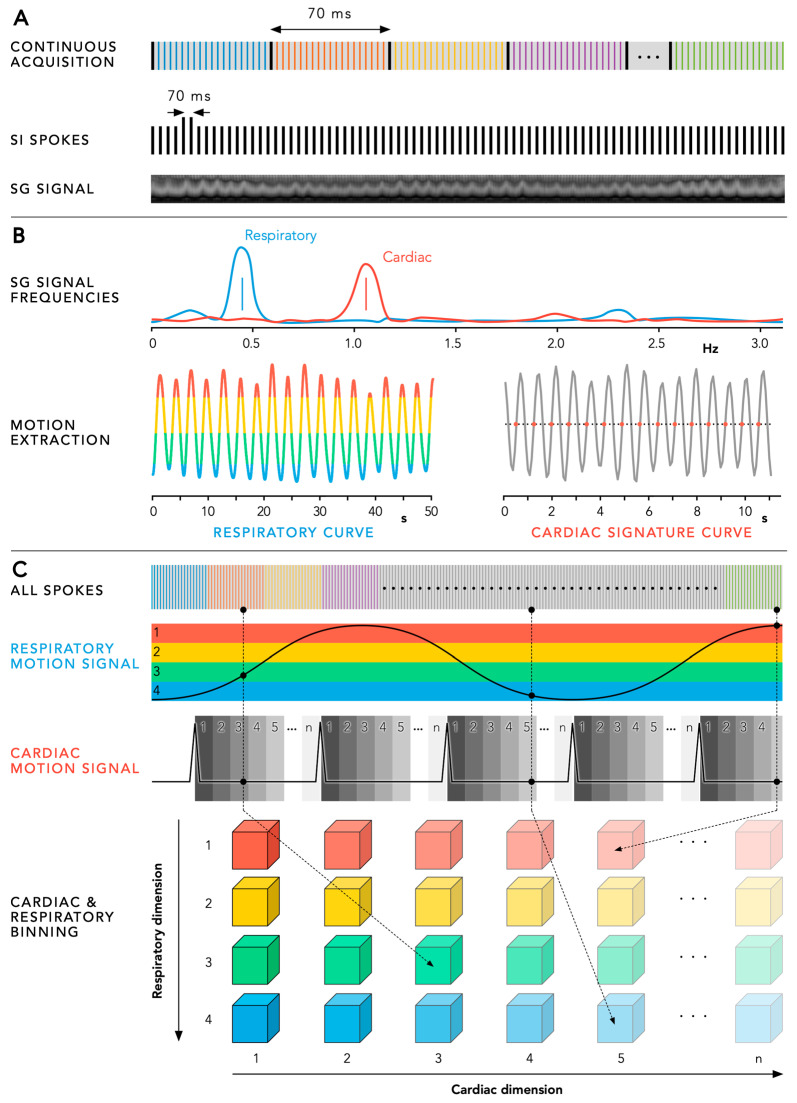
Panel (**A**) By extracting all superior-inferior (SI) spokes from each consecutive interleave, a self-gating (SG) signal is obtained. Panel (**B**) Using principal component analysis and filtering of this SG signal, the main respiratory and cardiac frequencies can be derived, and the respiratory and cardiac signature curves can be extracted. Panel (**C**) Using the respiratory and cardiac motion signals, all acquired radial spokes can be retrospectively binned into a variable number of cardiac phases and respiratory motion states. Using a compressed-sensing reconstruction, a 3D volume is obtained for each bin, ultimately leading to a 5D cardiac and respiratory motion-resolved dataset.

All imaging data are processed using a previously reported fully automated motion extraction and reconstruction framework in MATLAB (Mathworks, Natick, MA, USA). First, the SI spokes of each phyllotaxis interleave are collected to obtain an SG signal (Figure 4A). By using principal component analysis (PCA) of this SG signal, the main respiratory and cardiac frequencies are derived, while respiratory and cardiac signature curves are extracted (Figure 4B). Using this cardiac motion signal, the imaging data are retrospectively sorted into a variable number of cardiac phases depending on the subject’s heart rate (standard cardiac bin width of 50 ms). Simultaneously, the same imaging data are also subdivided over four equally populated respiratory motion states using the extracted respiratory motion signal. As a result, all acquired radial spokes are assigned to their respective cardiac phase and respiratory motion state, independently from their position in the phyllotaxis interleave, to maximize the reconstructed temporal resolution (Figure 4C).

Following the SG signal extraction and data sorting, a CS algorithm that exploits sparsity along both the cardiac and respiratory dimensions is used to reconstruct 5D (x-y-z-cardiac-respiratory) motion-resolved images [11,24]. An alternating direction method of multipliers algorithm with a total of 10 iterations is used to solve the CS optimization problem. The regularization weights along both the cardiac and respiratory dimensions can be chosen manually; however, they are often conservatively set to 0.001 to avoid motion compression artefacts while minimizing residual aliasing for optimal image quality. The entire reconstruction of the acquired free-running data, as mentioned above, is performed using a single mouse click without further user interaction.

**Figure 5:**
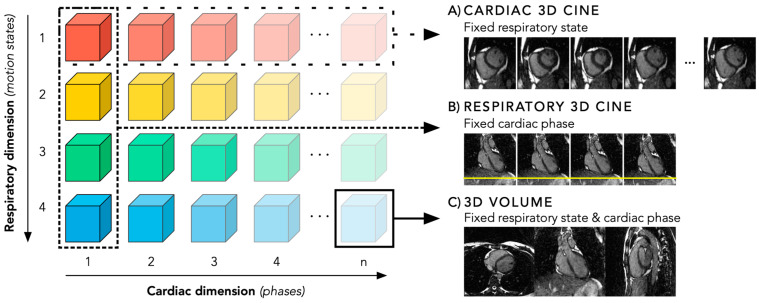
The obtained 5D image dataset can be visualised in various ways. (**A**) By fixing a specific respiratory motion state and looping through the cardiac phases, a cardiac 3D cine image is obtained (x-y-z-cardiac). (**B**) By fixing a specific cardiac phase and looping through the respiratory motion states, a respiratory 3D cine image is obtained (x-y-z-respiratory). (**C**) By fixing both a specific cardiac phase and respiratory motion state, a static 3D volume is obtained (x-y-z). For both dynamic 3D cines (**A**,**B**) and the static 3D volume (**C**), every desired (double) oblique cardiac view can be retrospectively selected using a multiplanar reconstruction.

After reconstruction, a 5D image dataset is obtained that can be exploited in various ways. First, one can select a specific respiratory motion state (e.g., end-expiration) and loop through the cardiac phases to obtain a 3D cine image in the cardiac dimension (x-y-z-cardiac, Figure 5A). Secondly, one can select a specific cardiac phase (e.g., end-diastole) and loop through the respiratory motion states to obtain 3D cine images in the respiratory dimension (x-y-z-respiratory, Figure 5B). Finally, one can select both a specific cardiac phase and respiratory motion state to obtain a static 3D volume (x-y-z, Figure 5C). Supplemental Video S1 shows an animated overview of the abovementioned three viewing methods. For both the 3D static and 3D cine images, the isotropic spatial resolution allows for excellent MPR opportunities. This enables the reader to select any desired (double) oblique cardiac view in the 3D volume, even while looping through the cardiac phases or respiratory motion states in case of 3D cine images, with a slice thickness that is only a fraction of the 8–10 mm in conventional 2D imaging.

### 3.3. Key Advantages

As already briefly discussed, conventional cardiac MRI relies heavily on a reliable ECG signal for prospective or retrospective gating to ’freeze‘ cardiac motion, and on repetitive breath-holding or inefficient respiratory navigators to eliminate respiratory motion. If these requirements are not sufficiently met, the resulting images may become blurred and show motion artefacts, potentially making them unsuitable for clinical decision-making. Accurate R-wave detection in the ECG signal is regularly hindered by unwanted overlaid signals from the magnetohydrodynamic effect [25] and rapid gradient switching, in particular during continuous acquisitions such as cine imaging. Additionally, repetitive breath-holds can cause patient discomfort and fatigue, or may even be impossible for some patients. While diaphragmatic drift may occur during a breath-hold, consecutive breath-holds can also be inconsistent, leading to overlapping slices and functional measurement errors. Conversely, free-running cardiac MRI uses an SG sequence that extracts cardiac and respiratory motion signals directly from the acquired image data, eliminating the need for an ECG signal and repetitive breath-holds. This not only precludes ECG signal problems and improves patient comfort, but also speeds up and simplifies the preparation of patients before scanning.

Additionally, standard 2D cardiac imaging requires the time-consuming and proficiency-demanding planning of (double) oblique cardiac views before acquisition, all while the patient remains in the scanner without data being collected. This leads to low acquisition efficiency. Furthermore, as each operator plans these cardiac views slightly differently, operator variability may be introduced, potentially affecting the resulting quantitative measurements. Finally, all desired cardiac views must be predetermined when using conventional 2D cardiac imaging, as no additional views can be created later. In contrast, free-running cardiac MRI addresses all these challenges. It eliminates the need for double oblique planning as only a 3D acquisition volume needs to be positioned around the heart based on the initial survey/localizer scan. Any desired cardiac view can be generated afterwards using an MPR of the 3D image (for each cardiac phase and respiratory state). This feature not only provides tremendous freedom in analysing the images but allows for additional measurements that are often not planned on beforehand, such as assessing atrial volumes and coronary arteries. Free-running MRI enables cardiac imaging to be performed by any operator, and not just by those specialized or specifically trained in CMR.

Finally, when scanning paediatric patients, general anaesthesia and intubation are usually required to enable breath-holding [26]. This not only carries risks for paediatric patients but also requires the presence and supervision of a significant additional workforce of medical personnel [27,28]. When using the FRF, however, breath-holding is no longer required which obviates this time-consuming, costly, and potentially dangerous procedure [29].

## 4. Recent Improvements to the Free-Running Framework

In 2021, Roy et al. further improved the FRF by introducing focused navigation (fNAV) for 3D non-rigid respiratory motion correction [30]. Instead of only sorting data into multiple respiratory states and reconstructing respiratory resolved images using XD-GRASP, 3D non-rigid intra-bin motion correction of the data is performed. Using an autofocusing-based algorithm [31] that converts a 1D unitless respiratory signal derived from the SI spokes into displacement fields along all three spatial dimensions with physical units, a final 3D image is obtained that is regionally corrected for intra-bin respiratory motion. More recently, Falcão et al. extended the use of fNAV to an FRF adapted for visualizing and measuring blood flow [32].

Also in 2021, Heerfordt et al. introduced a similarity-driven multi-dimensional binning algorithm (SIMBA) as an alternative reconstruction technique [33]. By leveraging intrinsic similarities in the data instead of relying on physiological assumptions and targeting specific motion states, the continuously acquired data can be clustered to find a motion-consistent subset that enables a simple and much faster reconstruction of a single anatomical 3D image. This novel whole-heart image reconstruction method enables the reconstruction of a motion-suppressed image in less than a minute, compared to fully motion-resolved compressed-sensing reconstructions that may last several hours. More recently, SIMBA was further optimized by sharing redundant information in the clustering dimension and by incorporating inter-cluster motion compensation into the CS reconstruction [34].

In 2022, Falcão et al. proposed to integrate an alternative navigation system, called pilot tone (PT), into the FRF for MR image-independent motion detection [35]. This PT navigation system, which comes integrated into the chest coil array, transmits a continuous-wave radiofrequency signal into the magnet bore [36,37]. This signal, with a frequency outside that of the frequency band of the MR imaging signal, is then captured by the receiver coils after having been modulated by the underlying physiological motion of the subject. Similarly to the aforementioned MR data-driven SG approach of the FRF, both respiratory and cardiac signature curves can be extracted from this signal, but now completely independently from the image acquisition and with a much higher sampling rate. This offers the opportunity to decouple the k-space trajectory from SG signal reception and provides the freedom to explore alternative k-space trajectories.

Most recently, in 2024, Roy et al. presented the use of non-rigid deformation fields, also referred to as ‘motion fields’ for inter-bin compensation of respiratory motion in the FRF [38]. Given the substantial undersampling of the individual bins (for each cardiac phase and respiratory motion state), direct estimation of deformation fields between these bins from the motion-resolved data is not feasible. However, after collapsing the cardiac dimension and performing intra-bin motion correction using the aforementioned fNAV approach, 3D inter-bin motion between the individual respiratory motion states can be estimated using non-rigid image registration [39]. Simultaneously, the fNAV-corrected data are binned by the cardiac phase. Combined, intra-bin *corrected* 5D cardiac and respiratory motion-resolved data with inter-bin *compensation* are obtained. The inclusion of these non-rigid 3D deformation fields enables larger regularization weights during reconstruction [24], which reduces artefacts without incurring motion blur and may be exploited for higher resolution or shortened scan durations. Although this work investigated the use of motion fields for inter-bin correction of respiratory motion only, similar work, albeit unpublished yet, has been performed for inter-bin compensation of cardiac motion.

## 5. Clinical Applications

To translate and exploit the efficiency and simplicity of this free-running framework as an automated push-button solution for the clinic, a variety of cardiac applications have been investigated in the past 10 years. These include anatomical imaging and coronary angiography, functional cine imaging, flow imaging, and quantitative mapping, which will be discussed in this section.

### 5.1. Anatomical Imaging and Coronary Angiography

High-resolution imaging of the heart, its coronary arteries, and the great vessels plays an increasingly important role in the detection and assessment of cardiovascular diseases, such as coronary artery disease (CAD) and congenital heart diseases (CHDs) [40,41,42]. Although coronary angiography (CAG) using X-rays is the reference standard for assessing CAD, it is invasive, uses ionizing radiation, and poses a small risk of complications. Coronary magnetic resonance angiography (CMRA) is a potential alternative with several advantages, including its inherent soft-tissue contrast and lack of ionizing radiation [43].

Over the past 25 years, CMRA has seen significant advancements but is still primarily used in research at specialized academic centres only. Its main drawbacks are the time-inefficient data collection and susceptibility to motion-induced artefacts. CMRA data collection is limited to the brief window of coronary quiescence (~50–150 ms), typically in mid-diastole, using electrocardiogram triggering. Achieving adequate high-resolution volumetric coverage therefore requires a large number of cardiac cycles, necessitating a free-breathing acquisition with advanced respiratory motion compensation. The SG concept, and ultimately the fully automated FRF, enabled complete cardiac and respiratory motion-resolved whole-heart imaging.

Already in 2014, Coppo et al. evaluated coronary angiography and cardiac cine imaging capabilities using a simplified 4D version of the FRF (respiratory motion correction instead of resolving respiratory motion and without a CS reconstruction) [9]. When employed in nine healthy adult volunteers at 1.5 T with an isotropic spatial resolution of 1.15 mm^3^, coronary artery image quality was very similar to that of a standard ECG-triggered reference acquisition and good agreement was found for cardiac volumes and ejection fractions between 4D and standard 2D cine imaging. Although the signal-to-noise ratio (SNR) was lower given the lower steady-state magnetization level and undersampling penalty, the 4D approach provided information about coronary anatomy at multiple time points in the cardiac cycle, along with data on ventricular function.

**Figure 6:**
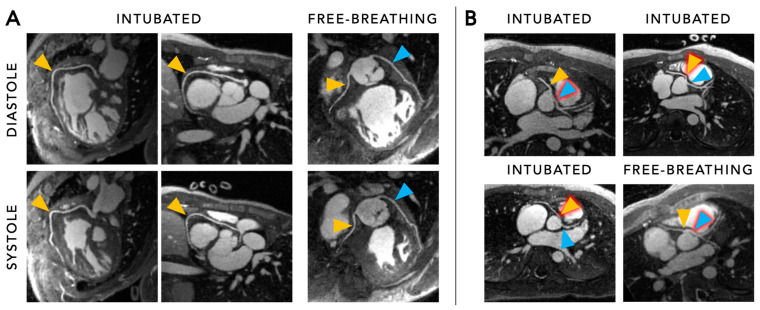
Panel (**A**) Diastolic and systolic coronary reformats of paediatric patients after ferumoxytol injection under general anaesthesia and intubation, and under sedation and free-breathing. Data were collected using the 5D free-running framework. The orange arrowheads indicate the right coronary artery (RCA), while the blue arrowheads indicate the left main (LM) and left anterior descending (LAD) coronary arteries. It can be observed that image quality and vessel conspicuity appear similar in the two indicated cardiac phases, as well as that image quality is comparable between intubated and free-breathing subjects. Panel (**B**) Coronary reformats for three intubated and one free-breathing paediatric subjects for simultaneous visualisation of the RCA ostium (orange arrowheads) and LM artery ostium (blue arrowheads). The arrowheads with a red glow highlight anomalous coronary vessel anatomy. Figure created using the original source images by Roy et al. [29] with author permission.

In 2022, Roy et al. investigated the role of the 5D cardiac and respiratory motion-resolved FRF for coronary angiography in paediatric CHD patients on a 1.5 T scanner [29]. All patients were administered ferumoxytol, an MRI contrast agent that circulates in the bloodstream for an extended period compared to standard gadolinium-based contrast agents [44]. Ferumoxytol therefore allows an extended image acquisition time which supports imaging with high spatial and temporal resolution which is particularly advantageous for paediatric patients with small anatomical structures and rapid heart rates [45]. Contrast-enhanced CMRA using the FRF was successfully employed and provided an excellent definition of cardiac anatomy—including that of the coronary arteries—in both intubated and free-breathing paediatric CHD patients (Figure 6). This clinical study underlined the simplicity, efficiency, and feasibility of contrast-enhanced CMRA using the FRF in paediatric CHD patients and further paved the way for its introduction into clinical workflows.

### 5.2. Functional Cine Imaging

Since the FRF is fully motion-resolved in both the cardiac and respiratory dimensions, 3D images of every cardiac phase or respiratory motion state can be reconstructed. Typically, the cardiac cycle is segmented into cardiac phases with a 50 ms bin width, while the respiratory motion is divided into four motion states. However, the FRF with its CS reconstruction has the capability of retrospectively and flexibly selecting the temporal resolution for image reconstruction. After reconstructing a static 3D image for each cardiac phase and motion state, multiple static 3D images can be played after each other to create a 3D cine image (i.e., x-y-z-cardiac). Although this can be performed in both the cardiac and respiratory dimensions, the former is mostly used and widely recognized as cardiac cine imaging for the assessment of cardiac volumes and function. Given these powerful capabilities of the FRF, anatomical imaging, coronary angiography, and cardiac cine imaging can all be performed using a single MRI acquisition of 5–10 min, depending on the required spatial resolution, without ECG and during free-breathing.

**Figure 7:**
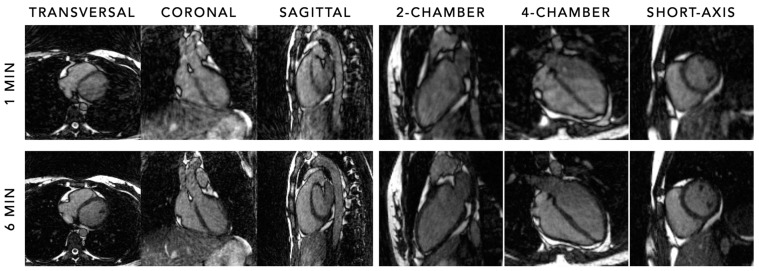
Cardiac cine imaging in transversal, coronal, and sagittal views, and in left two-chamber, four-chamber, and short-axis views (using multiplanar reconstructions), acquired using the 5D free-running framework in a healthy adult subject using a 1-min and 6-min acquisition without contrast at 1.5 T. Although the significantly increased noise, lower image quality, and lower observer confidence for the 1-min acquisition are apparent, cardiac volumes and function were still comparable to reference standard 2D breath-hold cine imaging (not shown in this figure).

Recent work, although unpublished yet, focussed on how much the acquisition time for cardiac cine imaging using the FRF could be shortened while still obtaining comparable cardiac volumes and ejection fraction compared to reference standard 2D breath-hold cine imaging. When comparing acquisition durations between 1 and 6 min, durations as short as 1 min still led to comparable quantitative measures, albeit with significantly noisier images and therefore lower observer confidence (Figure 7). These results underline the importance of focusing on improving efficiency and obtaining comparable clinical measures, instead of only striving for the highest image quality. The FRF therefore holds promise as an alternative to standard 2D cine imaging to improve workflow efficiency, simplicity, and patient comfort.

### 5.3. Flow Imaging

Phase-contrast MRI is a well-established technique to visualize and quantify blood flow, and increasingly used in the diagnosis and management of cardiovascular diseases [46]. Using a bipolar gradient, degrees of phase shift are encoded which directly correlate with the velocity of the moving protons in the bloodstream [47]. While 2D phase-contrast MRI can map blood flow in a single 2D slice, 3D phase-contrast MRI, also known as 4D flow (x-y-z-cardiac), can quantitatively evaluate haemodynamics across an entire 3D volume [48,49]. This allows simultaneous assessment of flow in the cardiac chambers and multiple surrounding vessels [50]. Although 2D phase-contrast MRI is well-established and routinely used, 4D flow is still limited by long and variable imaging times because of the navigator technology used for respiratory motion artefact suppression. This not only leads to reduced imaging efficiency but also discards data acquired outside of end-expiration, thereby prohibiting the measurement of respiration-resolved flow dynamics. Especially in CHD patients, the respiratory effects on cardiopulmonary circulation may be of great clinical interest [51].

**Figure 8:**
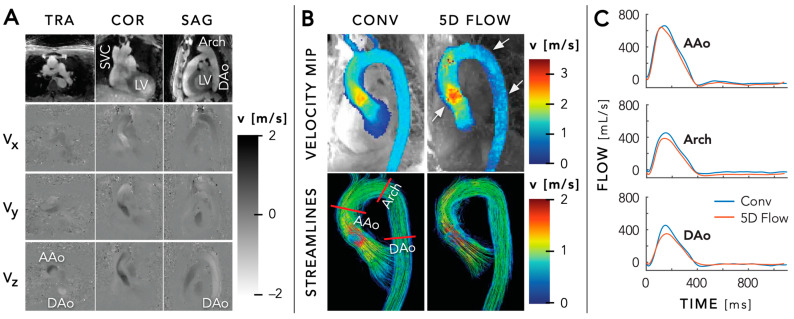
Panel (**A**) Magnitude images and phase-difference images for all three velocity-encoding directions (V_x_, V_y_, and V_z_) in a transversal (TRA), coronal (COR), and sagittal (SAG) view for a specific time point during systole of a 41-year-old man with bicuspid aortic valve disease. Panel (**B**) Peak systolic velocity maximum intensity projections (MIPs) and streamlines show good agreement between conventional navigator-gated 4D flow and free-running 5D flow imaging, with some overestimation in the ascending aorta (AAo) and underestimation in the arch and descending aorta (DAo) as indicated by the white arrows. Panel (**C**) Flow curves at three locations in the aorta, as indicated by the red lines in panel (**B**), demonstrate good agreement between the two techniques. The underestimation in the arch and DAo for 5D flow can be observed. LV = left ventricle, SVC = superior vena cava. Figure created using the original source images by Ma et al. [52] with author permission.

In 2020, Ma et al. expanded the existing FRF with bipolar gradients to enable cardiac and respiratory motion-resolved 3D flow imaging, referred to as 5D flow MRI (x-y-z-cardiac-respiratory, Figure 8) [52]. They validated its feasibility and performance in both in vitro and in vivo measurements. Although the in vitro results showed excellent agreement with conventional 4D flow-derived values, the in vivo results showed moderate agreement with conventional aortic 4D flow measurements. In 20 adult participants, 5D flow assessment demonstrated an overestimation in net flow and peak velocity up to 26% and 12%, respectively, in the ascending aorta, and an underestimation of <12% in the arch and descending aorta.

Later, in 2021, Ma et al. used this 5D flow FRF to explore the influence of arrhythmia on thrombogenic hemodynamic parameters in patients with atrial fibrillation (AF) [53]. Instead of using the fifth dimension for respiration, it was adapted to capture different RR interval durations in AF patients with arrhythmic heart rates to obtain RR-resolved 5D flow data (x-y-z-cardiac-RR interval duration). The RR interval durations were, similarly to respiration, divided into four bins, while no respiratory motion correction was used. The in vitro results demonstrated successful recovery of RR-binned flow curves compared to a real-time 2D phase-contrast MRI reference standard. In vivo results showed that AF burden was significantly correlated with 5D flow-derived peak and mean velocity and stasis measurements. This work demonstrated the feasibility of using an adapted RR-resolved 5D flow FRF to capture 3D haemodynamics in patients with AF in under 10 min, thereby offering a novel imaging approach for investigating arrhythmias.

### 5.4. Quantitative Mapping

Cardiac MRI can also be used to non-invasively and quantitatively measure fat concentration in the heart, which can assist in diagnosing pathologies characterized by the abnormal development of adipose cells [54,55]. Fat fraction quantification also has the potential to characterize the complex metabolic role of adipose tissues in obesity and diabetes, where increased amounts of epicardial, pericardial, and peri-coronary fat alter the cardiovascular disease risk profile [56,57]. Fat fraction quantification is typically performed using a multi-echo gradient-echo sequence that acquires images at different echo times [55].

**Figure 9:**
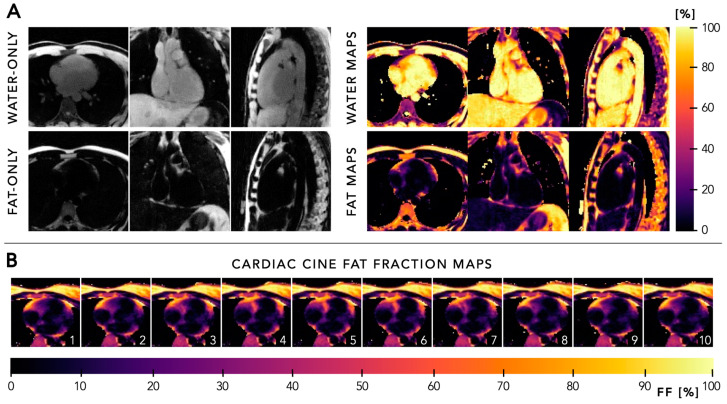
Panel (**A**) Water-only and fat-only images, and corresponding parametric maps of water fraction and fat fraction of a healthy adult subject as obtained using the proposed 6D free-running framework (x-y-z-cardiac-respiratory-echo) at 1.5 T. Panel (**B**) Cardiac fat fraction maps in a transversal view for each phase of the cardiac cycle during end-expiration as obtained using the proposed 6D free-running framework in a healthy adult subject at 1.5 T, allowing tracking of the displacement of pericardial fatty regions throughout the cardiac cycle. Figure created using the original source images by Mackowiak et al. [58] with author permission.

In 2023, Mackowiak et al. extended the FRF to 6D to enable multi-echo sampling and multi-peak fitting for fat–water separation and quantification at 1.5 T (x-y-z-cardiac-respiratory-echo, Figure 9) [58]. The aforementioned PT technique was added to obtain cardiac and respiratory signals [36,37]. In vivo results in 10 healthy volunteers demonstrated the feasibility of pericardial fat imaging and quantification throughout the cardiac cycle, and the motion-resolved end-diastolic 3D fat fraction maps showed a good correlation with ECG-triggered measurements (bias of −1.1%).

Later in 2024, Daudé et al. further built upon the work of Mackowiak et al. and evaluated it on 3 T [59]. Although benefitting from increased signal-to-noise to achieve higher spatial resolution, gradient correction using the gradient impulse response function (GIRF) was implemented to overcome distortions between odd and even echoes [60,61]. Their in vitro results in a water-fat phantom showed that fat fraction quantification was reliable and accurate using bipolar readout gradient with the GIRF correction, while it suffered from artefacts without correction (bias of 0.4% vs. 4.9%). In vivo measurements in 10 healthy volunteers and three diabetic patients demonstrated a significantly lower fat fraction of epicardial adipose tissue compared to subcutaneous fat. These studies showed that fat-fraction mapping using the FRF is feasible at both 1.5 and 3 T, enabling the investigation of epicardial adipose tissue in metabolic diseases.

Apart from fat-fraction mapping, quantitative T_1_ and T_2_ mapping in a free-running approach have been proposed [18,62,63,64,65]. However, since a different framework and different acquisition strategy were used for these, we will not further discuss these in this review.

## 6. General Recommendations

After addressing the clinical applications, it is important to consider several technical aspects that may influence the imaging outcomes of the FRF. These aspects include fat suppression, field strength dependency, and the role of contrast agents. Each of these factors plays a pivotal role in optimizing cardiac imaging outcomes using the FRF and will therefore be briefly discussed in this section.

### 6.1. Fat Suppression

In radial imaging, the fat signal should be suppressed as much as possible in case fat information is not of interest, as it helps to minimize strong streaking artefacts and image blurring caused by fat-water chemical shift. Fat suppression in MRI typically begins with a fat suppression preparation module that suppresses the MRI signal from fat tissue. As the fat signal recovers over time, multiple k-space lines can be collected. In Cartesian sampling, k-space sampling can be arranged so that the k-space centre is sampled when the fat signal is fully nulled following the fat suppression preparation. Although some k-space lines will always be sampled with a residual fat signal, these measurements can be deliberately acquired in the outer k-space regions, reducing their influence on image contrast. However, when using radial imaging, the performance of fat suppression can be compromised due to the repeated sampling of the k-space centre. Consequently, all k-space measurements, whether with or without fat signal, equally contribute to the final image. This prevents effective fat suppression in radial MRI and necessitates more advanced suppression strategies. Although water excitation techniques can be used, they are sensitive to magnetic field inhomogeneity at high fields, potentially causing unwanted signal loss. Recently, two fat suppression techniques for 3D radial imaging have been proposed, including lipid-insensitive binomial off-resonant radiofrequency excitation (LIBRE) [66] and fast interrupted steady-state (FISS) [67].

In 2019, Masala et al. implemented, optimized, and evaluated LIBRE [66], which uses two non-selective off-resonant rectangular radiofrequency sub-pulses, for fat suppression in the FRF [68]. Following parameter optimization using Bloch equations and in vitro validation, the optimized LIBRE pulses were evaluated for their use in the FRF in 20 adult subjects at 1.5 T. This LIBRE implementation was demonstrated to improve blood–fat contrast, vessel detection, and vessel sharpness when compared to other fat suppression methods, including water excitation and an interrupted free-running sequence using a chemically selective saturation (CHESS) fat saturation pulse. LIBRE thereby enabled superior fat suppression in the FRF without interrupting the 3D radial sequence, albeit with a penalty in scan time compared to its non-fat suppression counterpart.

**Figure 10:**
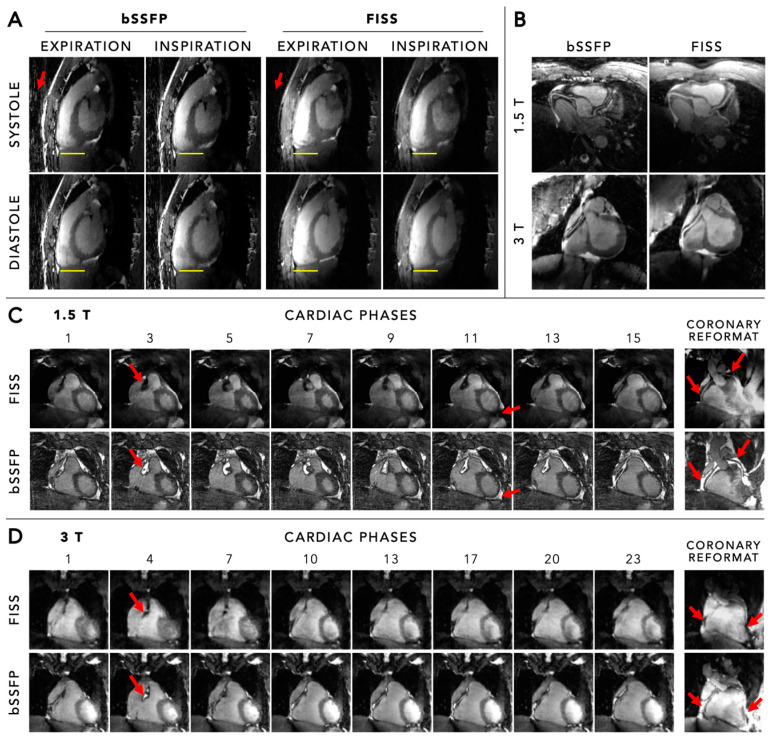
Panel (**A**) Comparison of anatomical images acquired by the 5D free-running framework using a standard balanced steady-state free-precession (bSSFP) and fast-interrupted steady-state (FISS) acquisition at 3 T. The 5D datasets were multiplanar reformatted to a sagittal view, and images are shown in systole and diastole at both end-expiration and end-inspiration. The yellow lines help to indicate respiratory motion. The red arrows indicate the decrease in streaking artefacts in FISS compared to bSSFP. Panel (**B**) Comparison of coronary reformats obtained using both bSSFP and FISS, at both 1.5 T and 3 T. The water–fat cancellation artefacts at the coronary vessel borders can be observed in the bSSFP images, while these are absent in the FISS images. Panel (**C**) Comparison of anatomical images using a bSSFP and FISS acquisition at 1.5 T. Both 5D image data were binned into 16 cardiac phases and four respiratory motion states, of which eight cardiac phases in the end-expiratory motion state are shown in coronal view for each method. The red arrows indicate cardiac regions containing fat that is suppressed using FISS but not using bSSFP. Panel (**D**) Comparison of anatomical images using a bSSFP and FISS acquisition at 3 T. Both 5D image data were binned into 26 cardiac phases and four respiratory motion states, of which eight cardiac phases in the end-expiratory motion state are shown in coronal view for each method. The red arrows indicate cardiac regions containing fat that is suppressed using FISS but not using bSSFP. Figure created using the original source images by Bastiaansen et al. [69] with author permission.

Another proposed fat suppression technique for 3D radial imaging is FISS, which has already shown its effectiveness in providing high SNR and blood–muscle contrast while simultaneously suppressing the fat signal under certain conditions when using bSFFP in 2D imaging [67]. In 2020, Bastiaansen et al. developed and evaluated the use of FISS in 3D radial imaging to implement fat suppression in the FRF [69]. After optimization using numerical simulations and phantom experiments, they evaluated fat suppression in volunteers at both 1.5 and 3 T. These results demonstrated a significantly decreased fat signal using FISS compared to standard bSSFP at both field strengths (Figure 10). With only a modest increase in scan time, high-resolution fat-suppressed 5D whole-heart imaging using the FRF could be performed, and FISS is currently the method of choice for fat suppression in the FRF.

### 6.2. Field Strength Dependency

Higher field strengths in MRI can offer improved signal-to-noise ratios and spatial resolution, albeit at the cost of increased susceptibility to artefacts. The FRF has been extensively evaluated at both 1.5 T and 3 T, demonstrating its flexibility at both clinically established field strengths. Although 3 T indeed brings additional signal-to-noise that can be exchanged for improved spatial or temporal resolution, imaging is more prone to artefacts. Most notable is the potential occurrence of banding artefacts when increased repetition times are used for bSSFP to incorporate, for example, bipolar gradients to enable flow imaging. Although GRE instead of bSSFP has been used to overcome these artefacts, GRE has reduced signal and blood–myocardium contrast compared to bSSFP and may partly diminish the obtained increase in SNR from moving to a higher field strength.

Apart from the clinically established field strengths of 1.5 T and 3 T, field strengths <1 T have recently gained considerable attention and are intensively being explored for cardiac imaging [70]. Staggering progress in hardware, software, and methodology has helped advance the hypothesis that this progress may revert the trend for ever-increasing field strength and be directed instead to making low-field systems more powerful, more cost-effective, and more widely accessible. Their reduced costs of purchase, installation, and maintenance, and advantages in system siting will facilitate the more global dissemination of MRI to the benefit of a larger patient pool and reach geographical regions that have not traditionally had easy access to MRI. This dissemination is further strengthened by the FRF as discussed in this review, which no longer requires highly trained MR operators and a relatively complex patient setup. The combination of low-field MRI and FRF technology therefore has an unprecedented potential.

**Figure 11:**
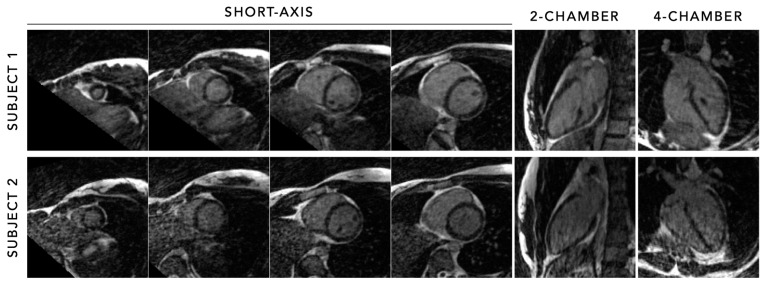
Cardiac cine imaging in various short-axis, and left two-chamber and four-chamber cardiac views (using multiplanar reconstructions), acquired using the 5D free-running framework in two healthy adult subjects using a 7 min acquisition without contrast at 0.55 T. Despite the significantly increased noise and decreased blood–myocardium contrast at low field compared to 1.5 T, cardiac anatomy and function can still be depicted in detail using the fully automated free-running framework without the need for an ECG signal or repetitive breath-holding.

Although unpublished yet, several preliminary studies have already demonstrated the feasibility of the FRF for anatomical, cine, and flow imaging at 0.55 T (Figure 11). The altered physics of low-field MRI are favourable for bSSFP imaging and are therefore directly advantageous for the FRF. Notwithstanding that SNR is significantly lower, novel reconstruction techniques and artificial intelligence (AI)-based methods can recoup part of the lost SNR compared to 1.5 T and 3 T, and thereby support field strengths <1 T as a valuable alternative to the more established field strengths.

### 6.3. Role of Contrast Agents

The role of contrast agents in cardiac MRI cannot be overstated [71]. These agents enhance tissue contrast and thereby aid in the detailed assessment of both the vasculature and myocardial tissue [72]. In the light of the FRF, the role of both gadolinium-based agents and ferumoxytol have been investigated. Although gadolinium-based agents are well-established and used in daily clinical routine cardiac MRI [73,74], their intravascular half-life is approximately 30 min and therefore makes them less suitable for long continuous acquisitions using the FRF. Ferumoxytol, on the other hand, has a much longer half-life of approximately 15 h, thereby achieving continuous and unaltered signal enhancement of the blood pool during longer acquisitions [44,45]. In addition, this longer half-life allows for injection before, instead of during, the MRI examination, thereby further improving efficiency and workflow. For these reasons, ferumoxytol is the preferred contrast agent when performing contrast-enhanced cardiac imaging using the FRF [29,34].

## 7. Future Perspectives

Despite all the advantages of the FRF, a broader penetration of this technology beyond academic centres has not happened thus far for the following reasons. First of all, CS reconstruction is time-consuming even on very powerful computer hardware, which adversely affects clinical utility where almost immediate image reconstruction is a requirement. However, this may be further addressed with AI-based algorithms and thorough validation. Secondly, image analysis software is currently not designed to accommodate 5D data, and while there is no product of a free-running approach on any vendor’s MRI system, there will not be an analysis software soon that can accommodate these 5D datasets. Coupled with that is the significant increase in data to be analysed: while a traditional cardiac 2D scan includes 10–12 short-axis slices of 5–10 mm each, the isotropic free-running dataset with an isotropic spatial resolution of 1–2 mm easily leads to a 5- to 10-fold increase in multiplanar reformatted planes that are available for analysis and can no longer be handled by a single operator in a reasonable amount of time. Therefore, AI-assisted algorithms are also needed here to make inroads. Finally, the utility and reproducibility of this approach will have to undergo deliberate and rigorous testing in larger multi-centric trials that include gold-standard comparisons.

## 8. Conclusions

Cardiac MRI using the fully automated free-running framework is an easy-to-use and powerful alternative to conventional approaches requiring ECG electrodes, repetitive breath-hold commands, or inefficient navigators, and time-consuming view planning. Given its clear advantages and continued improvements, it is expected to expand beyond academic centres. The implementation of this framework on more cost-effective low-field systems may further contribute to a broader global dissemination of cardiac MRI.

## Supplementary Materials

The following supporting information can be downloaded at: https://www.mdpi.com/article/10.3390/diagnostics14171946/s1, Video S1: An animated overview of three viewing methods for 5D cardiac and respiratory motion-resolved data. Top panel: By fixing a specific respiratory motion state and looping through the cardiac phases, a cardiac 3D cine image is obtained (x-y-z-cardiac). Middle panel: By fixing a specific cardiac phase and looping through the respiratory motion states, a respiratory 3D cine image is obtained (x-y-z-respiratory). Lower panel: By fixing both a specific cardiac phase and respiratory motion state, a static 3D volume is obtained (x-y-z). A fly-through in the transversal, coronal, and sagittal view is shown here. For both dynamic 3D cines (top and middle panel) and the static 3D volume (lower panel), every desired (double) oblique cardiac view can be retrospectively selected using a multiplanar reconstruction. Video created using the original source images by Roy et al. [40] with author permission.

## Abbreviations

AF	atrial fibrillation
AI	artificial intelligence
bSSFP	balanced steady-state free-precession
CAD	coronary artery disease
CAG	coronary angiography
CHD	congenital heart disease
CHESS	chemically selective saturation
CMRA	coronary magnetic resonance angiography
CS	compressed sensing
ECG	electrocardiogram
FISS	fast interrupted steady-state
fNAV	focused navigation
FRF	free-running framework
GIRF	gradient impulse response function
GRE	gradient echo
LIBRE	lipid-insensitive binomial off-resonant radiofrequency excitation
MPR	multiplanar reconstruction
MRI	magnetic resonance imaging
PCA	principal component analysis
PT	pilot tone
SG	self-gating/self-gated
SI	superior-inferior
SNR	signal-to-noise ratio
SIMBA	similarity-driven multi-dimensional binning algorithm
XD-GRASP	extra-dimensional golden-angle radial sparse parallel
